# Case report: composite pancreatic intraductal papillary mucinous neoplasm and neuroendocrine tumor: a new mixed neuroendocrine-non-neuroendocrine neoplasm?

**DOI:** 10.1186/s13000-021-01165-5

**Published:** 2021-11-20

**Authors:** Jingci Chen, Pengyan Wang, Ke Lv, Weixun Zhou

**Affiliations:** 1 Department of Pathology, Peking Union Medical College Hospital, Chinese Academy of Medical Sciences and Peking Union Medical College, Beijing, 100730 China; 2 Department of Ultrasound, Peking Union Medical College Hospital, Chinese Academy of Medical Sciences and Peking Union Medical College, Beijing, 100730 China

**Keywords:** MiNEN, Composite, *KRAS*, Case report

## Abstract

**Background:**

Mixed neuroendocrine-non-neuroendocrine neoplasms (MiNEN) of the pancreas are extremely rare. Their pathogenesis and molecular landscape are largely unknown. Here, we report a case of mixed pancreatic intraductal papillary mucinous neoplasm (IPMN) and well-differentiated neuroendocrine tumor (NET) and identify its genetic alterations by next-generation sequencing (NGS).

**Case presentation:**

A fifty-year-old male was admitted into the hospital for evaluation of a pancreatic lesion detected during a routine examination. Abdominal ultrasound indicated a hypoechoic mass of 2.6 cm at the head of the pancreas. Malignancy was suspected and partial pancreatectomy was performed. Thorough histopathological examination revealed a mixed IPMN-NET. In some areas, the two components were relatively separated, whereas in other areas IPMN and NET grew in a composite pattern: The papillae were lined with epithelial cells of IPMN, and there were clusters of NET nests in the stroma of papillary axis. NGS revealed shared somatic mutations (*KRAS*, *PCK1*, *MLL3*) in both components. The patient has been uneventful 21 months after the surgery.

**Conclusions:**

Our case provides evidence of a common origin for mixed IPMN-NET with composite growth features. Our result and literature review indicate that *KRAS* mutation might be a driver event underlying the occurrence of MiNEN. We also recommend the inclusion of mixed non-invasive exocrine neoplasms and neuroendocrine neoplasms into MiNEN.

**Supplementary Information:**

The online version contains supplementary material available at 10.1186/s13000-021-01165-5.

## Background

Mixed neuroendocrine-non-neuroendocrine neoplasms (MiNEN) of the pancreas are a heterogenous group of malignancies and are extremely rare. According to the 2017 World Health Organization (WHO) classification of tumors of endocrine organs and the 2019 WHO classification of digestive system tumors, MiNENs are carcinomas composed of both a non-neuroendocrine carcinoma and neuroendocrine neoplasm (NEN), each of which constitutes ≥30% of the neoplasm [1–4]. Most pancreatic MiNENs are mixed ductal-neuroendocrine carcinomas or mixed acinar-neuroendocrine carcinomas and both components are usually high-grade [5]. Although the current WHO definition requires that the non-neuroendocrine counterparts be invasive carcinoma, occasionally they can be solely carcinoma precursors [6]. Here, we present an extremely unusual case of mixed intraductal papillary mucinous neoplasm (IPMN) and well-differentiated neuroendocrine tumor (NET) with detailed discussion about its growth pattern.

The etiology of MiNEN is controversial and largely unknown. The current WHO category applies only to tumors in which two components are clonally related, but not to two collision tumors [4]. To understand the relationship between the two components and to explore the pathogenesis, we manage to separate them by macrodissection and perform next-generation sequencing (NGS) with a panel of 1021 genes. The presentation of this case and literature review would help elucidate the possible origin of mixed IPMN-NET.

## Case presentation

**Fig. 1:**
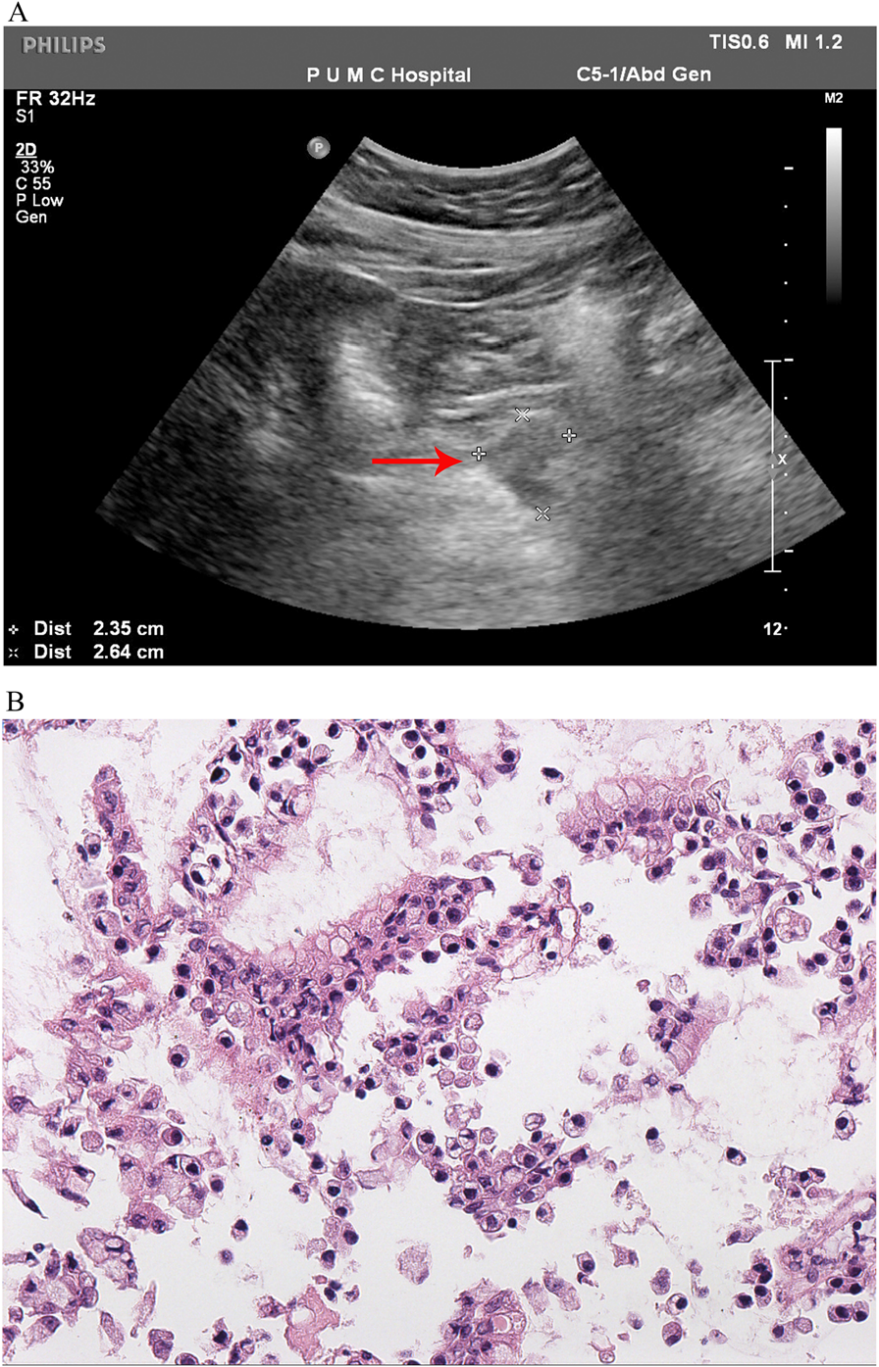
**A** Abdominal ultrasound showed a hypoechoic mass (red arrow) which tended to protrude outward at the head of the pancreas. (**B**) Cell block of fine needle aspiration showed mucinous columnar cells with mild dysplasia and scattered plasmacytoid cells with salt-and-pepper chromatin (HEx200)

A fifty-year-old male was admitted to the hospital for evaluation of a pancreatic lesion detected during a routine examination. Abdominal ultrasound revealed a hypoechoic mass of 2.6 cm at the head of the pancreas (Fig. 1A). The mass infiltrated into the surrounding adipose tissue and a pancreatic carcinoma could not be ruled out. The patient was asymptomatic and the serum tumor markers including CA-125, CA19–9, CA72–4, CEA, and AFP were all within normal limits. The patient had no medical or psycho-social history and no genetic tests were performed previously. Physical examination revealed no obvious abnormalities. Ultrasound-guided pancreatic fine needle aspiration was performed. On the cell block, the lesion was composed of mucinous columnar cells with mild dysplasia and scattered plasmacytoid cells with salt-and-pepper chromatin (Fig. 1B). Immunohistochemistry (IHC) showed AE1/AE3(+), chromogranin A (CgA) (+), synaptophysin (Syn) (+), and β-catenin (membranous +). Therefore, a mixed mucinous neoplasm and NEN was suspected.

**Fig. 2:**
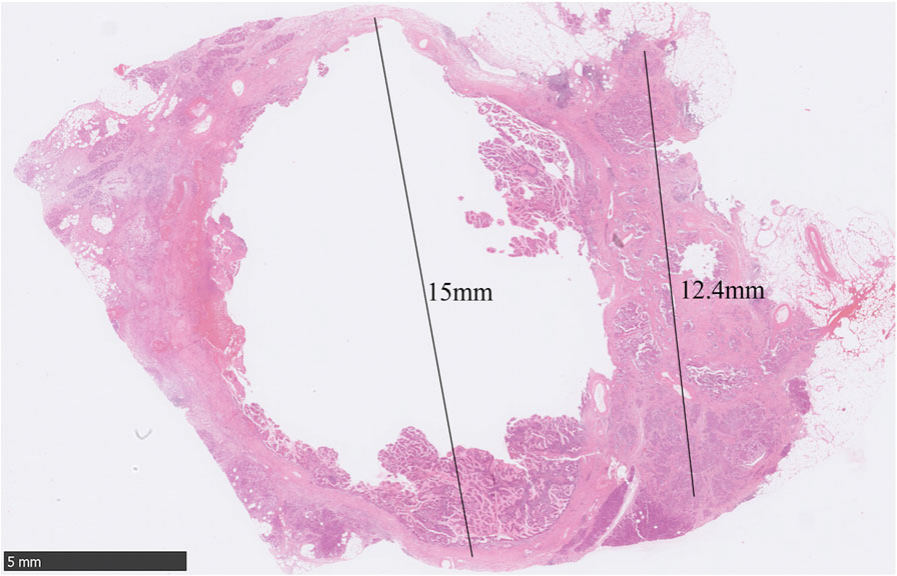
Whole-mount section of the lesion

**Fig. 3:**
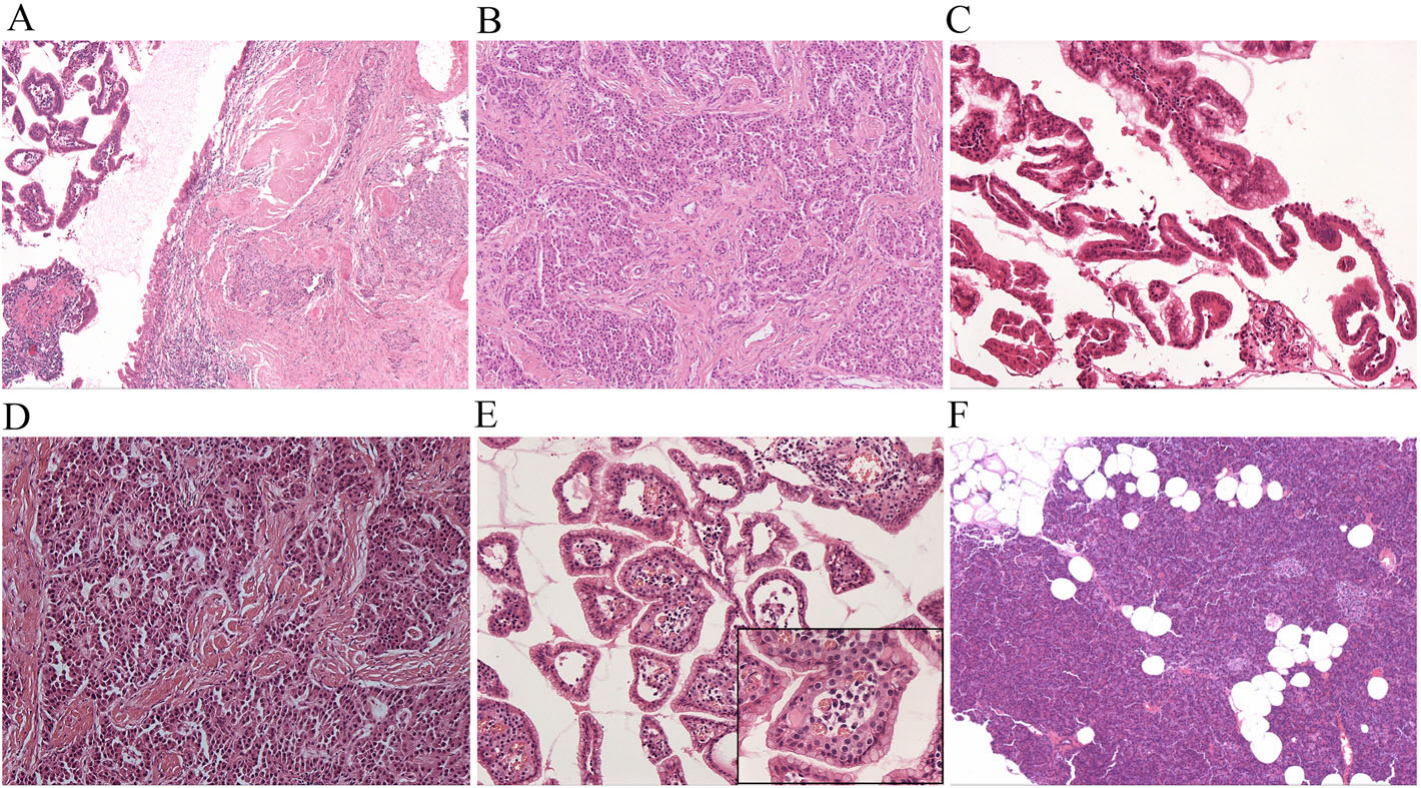
**A** Mixed areas of IPMN (left) and NET (right) components (HEx40). (**B**) Relative separate NET component (HEx40). (**C**) Relative separate IPMN component was composed of papillae covered by epithelial cells with mild to moderate dysplasia (HEx100). (**D**) Relative separate NET component showed the NET cells had round to oval nuclei. Few mitoses were found (HEx100). (**E**) In mixed areas, the papillae were lined with epithelial cells of IPMN, and filled with clusters of NET nests in the stroma (HEx100). Inset: Composite IPMN-NET. (**F**) Pancreatic tissue in the background was relatively normal (HEx40)

**Fig. 4:**
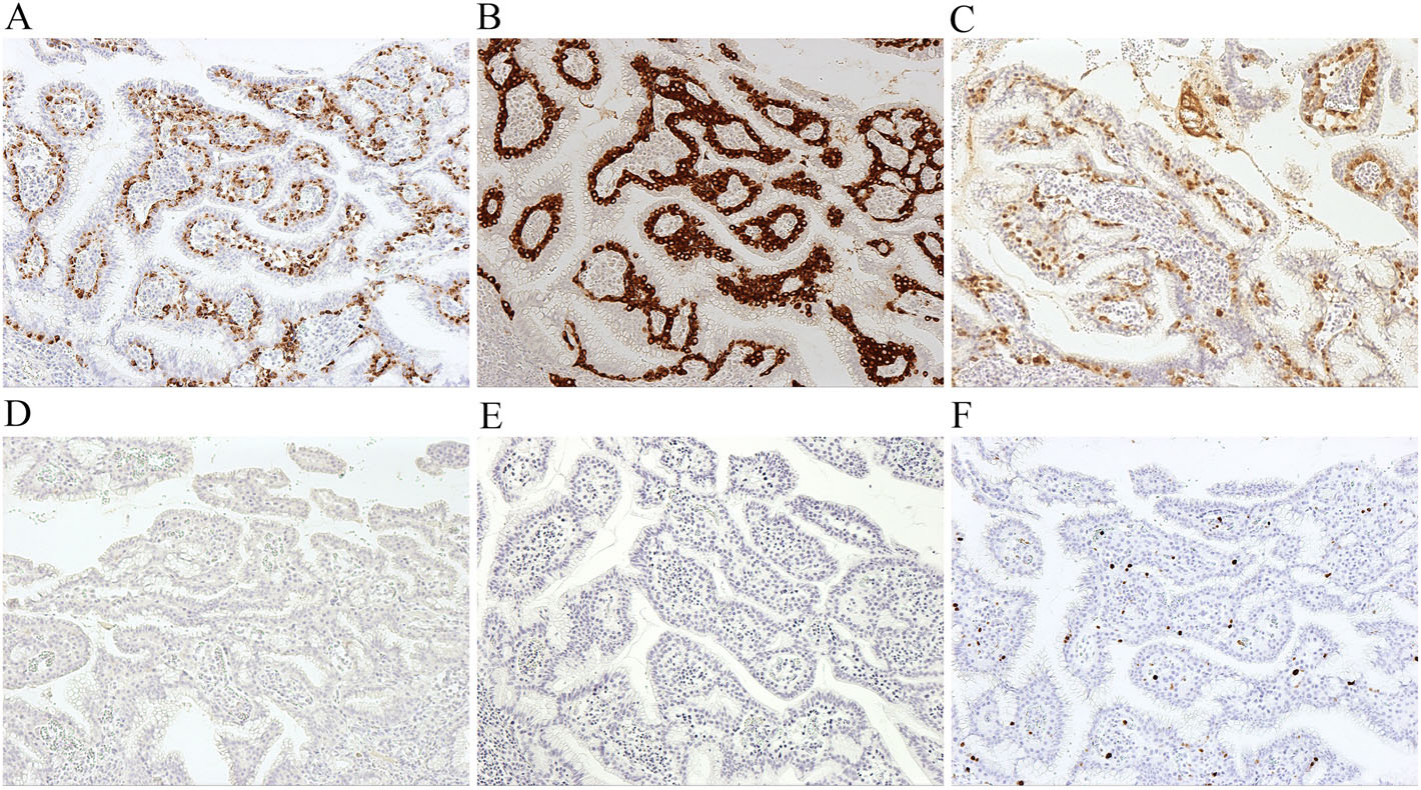
(**A**) NET component was positive for CgA (IHCx100). (**B**) NET component was strongly positive for Syn (IHCx100). (**C**) NET component was positive for glucagon (IHCx100). (**D**) NET component was negative for insulin (IHCx100). (**E**) NET component was negative for somatostatin (IHCx100). (**F**) Composite IPMN-NET showed a low Ki-67 index (IHCx100)

Partial pancreatectomy was performed. Grossly, the specimen measured 5.3 × 4.0 × 3.3 cm. On the cut surface, a cystic lesion with rough inner surface was found. The cyst measured 1.5 cm (Fig. 2). Careful histological examination revealed two components: IPMN and neuroendocrine components (Fig. 3). In some areas of the cyst, there was a classical IPMN component characterized by papillae forming and intraductal proliferation of columnar mucin-producing cells. Neuroendocrine components measured about 1.2 cm and could be found along the wall of the cyst, within the pancreatic tissue, as well as inside of the stroma of papillae. It grew in an organoid or trabecular pattern and partially infiltrated into the surrounding tissue, consistent with the imaging features. The nuclei were round to oval, with fine (salt-and-pepper) chromatin. The cytoplasm was finely granular and slightly eosinophilic. Positive staining for CgA and Syn confirmed the neuroendocrine differentiation (Fig. 4A, B). IHC for various hormones were also performed and the neuroendocrine components showed positive for glucagon and negative for gastrin, insulin, and somatostatin (Fig. 4C-E). The background pancreatic tissue was nearly normal: The structure of lobules was clear. Little acute or chronic inflammation, fibrosis, or acinar atrophy was observed (Fig. 3F). There was no other IPMN, mucinous cystic neoplasm (MCN), or pancreatic intraepithelial neoplasia (PanIN). The number, shape and size of islets were normal.

For the neuroendocrine components, an NET rather than neuroendocrine hyperplasia (islet hyperplasia) was considered for several reasons: First, the lesion was relatively isolated and the background pancreatic tissue was normal. There was no increased number or size of islets and their shape was regular. The β-cells showed normal nuclei without enlarged or hyperchromatic characteristics. In contrast, neuroendocrine hyperplasia more commonly had multiple atypical β-cells, increased number and size of islets, irregular islet shape, and sometimes patients could have history of chronic pancreatitis, carcinoma, etc. Second, the diameter of the lesion (1.2 cm) was even larger than the cut-off value of pancreatic neuroendocrine microadenomas (0.5 cm). Third, the lesion was diffusely positive for only one hormone, whereas neuroendocrine hyperplasia was usually composed of several types of endocrine cells. In addition, clinically, the latter was more commonly related to hyperinsulinemic hypoglycemia rather than α-cell hyperplasia with or without hyperglucagonemia.

Notably, in some areas of the cyst, the two components were highly intermingled and grew in a “truly mixed” pattern. The papillae were lined with epithelial cells of IPMN, and there were clusters of NET nests in the stroma (Fig. 3E). The whole lesion was composed of approximately 60% of IPMN component and 40% of NET. No lymph node metastasis, angioinvasion, or perineural spreading was found.

Each component was graded according to the 2019 WHO classification of digestive system tumors ^4, 5^. For IPMN, the architecture of papillae was simple and cell nuclei were mild to moderate atypia, mostly located at the base of the cells. Few mitoses were found. Thorough sampling of the lesion revealed no invasive carcinoma. Therefore, it was graded as IPMN with low-grade dysplasia. For NET, HE staining revealed mild nuclei atypia, no necrosis, and few mitoses (< 1/10HPFs). Ki-67 index by IHC was 2% (Fig. 4F). Therefore, the NET was graded as G1. Our final diagnosis was mixed IPMN-NET.

We further explored the molecular changes of this tumor. The two components were separated by macrodissection. Each component contained a minimum of 60% of tumor. DNA was extracted from formalin-fixed paraffin-embedded tissue. Genomic DNA were sheared into 200-bp to 250-bp fragments using a Covaris S2 instrument (Woburn, MA, USA), and indexed NGS libraries were prepared. All libraries were hybridized to self-built probes and sequencing was performed using the MGISeq-2000 Sequencing System (BGI, Shenzhen, China) according to the manufacturer’s guidelines with a panel of 1021 cancer-related genes (Supplementary table). The average sequencing depth was 655x. Single nucleotide variants (SNV), insertions or deletions (INDEL), gene fusions, and copy number variations (CNV) were analyzed and several point mutations in *KRAS*, *PCK1*, *MLL3* were identified (Table 1). The tumor mutation burden (TMB) in both components was low: 0.96Muts/Mb in IPMN component and 0.96Muts/Mb in NET component. No INDEL, gene fusions or CNV were detected. The mutation status of *PCK1* and *MLL3* in pacreatic neoplasms was searched in Catalogue Of Somatic Mutations In Cancer (COSMIC) database and provided in Table 2. No germline mutations were detected.

**Table 1:** Genetic alterations of our case and comparison with pure neoplasms

Gene	Our case		Reported altered genes in pure neoplasms	
	IPMN component	NET component	IPMN	NEN
*KRAS* (MAF)	NM_033360.2 Exon 2, p.G12V (6.1%)	NM_033360.2 Exon 2, p.G12V (4.7%)	*GNAS*, *KRAS*, *RNF43*, *KLF4*, *NBPF1*, *CDKN2A*, *APC*, *TP53*, *CTNNB1*, *RMB10*, *BSN*, *SETBP1*, *AJAP1*, *IGSF3*, *USP6*, *STK11*, etc. [7, 8]	*MEN1*, *DAXX*, *ATRX*, *TSC2*, *PTEN*, *PIK3CA*, *TP53*, *RB*, *VHL*, *MUTYH*, *BRCA2*, *CHEK2*, *ARID1A*, *BCOR*, *CDNK1B*, *KRAS*, *SMAD4*, *YY1*, *MLL3*, etc. [9–12]
*PCK1* (MAF)	NM_002591.3 Exon 14, p.P605H (3.2%)	NM_002591.3 Exon 14, p.P605H (3.0%)		
*MLL3* (MAF)	NM_170606.2 Exon 10, p.V649M (3.0%)	NM_170606.2 Exon 10, p.V649M (2.5%)		

*MAF* mutant allele fractions.

**Table 2:** Somatic mutations of *PCK1* and *MLL3* in pancreatic neoplasms in COSMIC database

Genes	CDS Mutation	Pancreatic neoplasm subtypes
*PCK1*	c.410G > A, c.865G > A, c.965A > G, c.992G > A, c.1095C > T, c.1187-21G > A, c.1391C > T, c.1656C > G, c.*369G > A, c.*371G > A, c.*373G > A, c.*1678A > G	Ductal carcinoma; some samples with unknown histology subtype
*MLL3*	c.568C > T, c.754C > A, c.1017G > C, c.1042G > A, c.1076G > A, c.1131A > T, c.1139G > T, c.1174C > T, c.1304G > A, c.2183A > C, c.2185A > G, c.2198del, etc.	Ductal carcinoma; PanIN; NET; acinar carcinoma; dysplasia-in situ neoplasm; sarcomatoid carcinoma; samples with unknown histology subtype

*CDS* coding DNA sequence.

Currently, the patient is uneventful at 21 months of regular follow-up.

## Discussion

We describe a mixed IPMN-NET pathologically characterized by both “truly mixed” pattern and relatively separated pattern. Although the clinicopathological features of concomitant IPMN and NEN have been described, researchers have not paid much attention to their growth pattern, which might have relationship with their pathogenesis. We summarize previously reported cases and classify them according to the morphological classification by de Mestier et al. (Table 3) [3]. Regrading of IPMN and NEN is based on the 2019 WHO classification of digestive system tumors and 2017 WHO classification of tumors of endocrine organs. Literature review of their pathological features demonstrates that most of them are concurrent or collision (Case 1–14 in Table 3), which should not be considered as MiNEN. A truly composite IPMN-NEN is extremely rare (Case 31–33 in Table 3). Notably, although Case 16 partially grew in a way which could not be classified clearly, further cytogenetic analysis confirmed their collision pattern [21].

**Table 3:** Literature review of 33 cases of concomitant IPMN and NEN

Case No.	IPMN grading	NEN grading	Morphological classification	Molecular studies	Follow-up	Ref
1	High	NA	Concurrent	NA	NA	Marrache et al., 2005 [13]
2–6	Low	NA	Concurrent	NA	NA	Marrache et al., 2005 [13]
7	Invasive	NA	Concurrent	NA	Uneventful 10 months after the surgery	Goh et al., 2006 [14]
8	Invasive	NA	Concurrent	NA	Uneventful 70 months after the surgery	Goh et al., 2006 [14]
9	Low	NA	Concurrent	NA	Uneventful 5 months after the surgery	Goh et al., 2006 [14]
10	NA	G1	Concurrent	NA	NA	Tewari et al., 2013 [15]
11	Low	G1	Concurrent	NA	NA	Ishida et al., 2013 [16]
12	Low	NA	Collision	NA	Uneventful	Mortele et al., 2009 [17]
13	Low	G1	Collision	NA	Uneventful 18 months after the surgery	Kadota et al., 2013 [18]
14	Invasive	G2	Collision	NA	Uneventful 5 years after the surgery	Ishizu et al., 2016 [19]
15	High	NEC	Collision or composite	NA	Dead 10 months after the surgery	Stukavec et al., 2007 [20]
16	Invasive	G2	Collision or composite	Cytogenetic analysis of LOH	NA	Moriyoshi et al., 2013 [21]
17–20	Low	NA	NA	NA	NA	Gill et al., 2009 [22]
21	NA	NA	NA	NA	Uneventful for 1 year (follow up without surgery)	Larghi et al., 2009 [23]
22	NA	NA	NA	NA	Uneventful	Larghi et al., 2009 [23]
23	Invasive	NEC	NA	Testing for *KRAS*	NA	Tewari et al., 2013 [15]
24	High	NEC	NA	Testing for *KRAS*	NA	Tewari et al., 2013 [15]
25–29	NA	NA	NA	NA	NA	Sahora et al., 2016 [24]
30	NA	NA	NA	NA	Uneventful for 1 year (follow up without surgery)	Costa et al., 2017 [25]
31	Invasive	NEC	Composite	NA	Dead 6 months after the surgery	Hashimoto et al., 2008 [26]
32	High	G1	Composite	Direct sequencing of *KRAS*	Uneventful 1 year after the surgery	Igarashi et al., 2019 [27]
33	High	G2	Composite	NGS; FISH for *CCND1*	Uneventful 27 months after the surgery	Schiavo Lena et al., 2020 [6]
Our case	Low	G1	Composite	NGS	Uneventful 21 months after the surgery	

*NA* not available, *LOH* loss of heterozygosity.

Concurrent is defined as two components detected at different sites but at the same time.

Mixed non-endocrine and endocrine components in digestive system tumors have been described early [28–31]. It was considered that most cases contained at least adenocarcinoma and the 2010 WHO classification used “mixed adenoneuroendocrine carcinoma” (MANEC) to describe them [32–34]. However, the previous term did not cover all the possibilities, and the 2019 WHO has replaced it with MiNEN [2, 4]. Notably, neoplasms whose non-neuroendocrine components are solely preinvasive lesions are still excluded [4]. Another term has been proposed to describe these subtypes: “Mixed adenoneuroendocrine tumor” (MANET) is used to underline an indolent group of tumors composed of adenoma and well-differentiated NET [35, 36]. Based on previous research, MANET is more commonly used and discussed in stomach, colon, and rectum [35, 37–39]. However, similar situations happen in pancreas and a mixed IPMN-NET also theoretically fulfills the requirements of mixed “adenoma” and well-differentiated NET. No mixed intraductal tubulopapillary neoplasm (ITPN)-NEN, mixed intraductal oncocytic neoplasm (IOPN)-NEN, or mixed MCN-NEN has been reported. Only rare cases of MCN with associated invasive carcinoma and neuroendocrine component have been reported [40].

The current definition of MiNEN also requires that the two tumor components be a single tumor of common origin rather than a collision of two tumors. The two components in our case are not only morphologically intermingled, but also have similar molecular changes. Our case and literature review demonstrate that the entity of two well-differentiated, clonally related components does exist, and the non-neuroendocrine components could be non-invasive [3]. Based on this fact and the miscellaneous terms that have been used (MiNEN, MANEC, MANET), we recommend that the subgroup of non-invasive exocrine neoplasms as non-neuroendocrine counterparts (including MANET) be included in MiNEN. We also recommend that the two components should be reported and graded separately in clinical practice, since different components could have different prognosis (Table 3) [41, 42]. In our case, the clinical behavior is relatively benign, which is consistent with other composite IPMN-NET and further supports the heterogeneity of outcome depending on the histological subtypes [6, 26, 27]. Another point that needs to be kept in mind is the differential diagnosis between NET and neuroendocrine hyperplasia. The latter has a series of major and minor critieria and its diagnosis, especially diffuse hyperplasia, might need near total pancreatectomy [43–45].

The pathogenesis of concomitant IPMN and NEN is controversial. Four hypotheses have been proposed. The first is collision of IPMN and NEN [14]. The second is transdifferentiation of IPMN cells into NEN cells. The third is transdifferentiation of NEN cells into IPMN cells [13, 27]. The fourth hypothesis is that pancreatic progenitor cells differentiate into both NEN and IPMN cells [27]. Based on histopathology, the hypothesis of transdifferentiation seemed unlikely when IPMN and NEN happened in different sites, which is the condition in most of the cases (Table 3). However, deeper investigation is lacking and not much research has demonstrated the genetic alterations of concomitant IPMN-NET. In 2013, Moriyoshi et al. firstly proved the hypothesis of collision by cytogenetic analysis of LOH in a patient with multiple endocrine neoplasia type 1 [21]. Tewari et al. performed molecular testing for *KRAS* in two cases of concomitant IPMN (invasion in one case) and NEC and both were wild-type [15]. Currently, only 2 cases of mixed IPMN-NET with typical composite morphology included molecular studies (Table 3) [6, 27]. Igarashi et al. first revealed the presence of *KRAS* (p.G12V) mutation in IPMN and transitional areas rather than NET area, supporting the fourth hypothesis [27]. Schiavo Lena et al. detected *KRAS* (p.G12D), *GNAS* (p.R201H), *CDKN2A* (p.Y44*) mutations and *CCND1* amplification (copy number 28) in both IPMN and NET components, serving as an evidence of a common cell origin [6]. Based on the currently limited data, our study used broad panel NGS instead of focusing on a few genes and provided the molecular landscape of composite IPMN-NET. We also identified *KRAS* mutation in both components. Combining the results, *KRAS* mutation might be an important diver mutation of composite IPMN-NET. Since the status of *KRAS* have been studied in pure IPMN and NEN in multiple studies, we further searched COSMIC database for the information of the other two mutated genes (*PCK1* and *MLL3*) in pancreatic neoplasms (Table 2) [46–48]. We found that most *PCK1* mutations occur in pancreatic ductal adenocarcinoma (several samples with unknown histology subtype). The specific *PCK1* p.P605H (c.1814C > A) mutation has only been reported in squamous cell carcinoma in the lung (sample name: TCGA-70-6722-01). For *MLL3*, its mutation has been reported in various pancreatic lesions including NEN and simple mucinous cysts, but not in IPMN (Tables 1,2) [49]. Intertestingly, it has also been detected in neuroendocrine components in one gastric MiNEN [50]. However, *MLL3* p.V649M (c.1945G > A) has never been reported. Our result suggests there might be other unknown mechanisms underlying composite IPMN-NET. A comparison between mixed IPMN-NET and its pure tumor counterparts is also shown in Table 1. According to Schiavo Lena et al., mixed IPMN-NET seems to share more genetic changes with IPMN [6]. However, our result favors the unique molecular alterations in composite IPMN-NET. Additionally, the same mutation spectrum in two components supports the hypothesis of one common origin.

Our study has limitations: Contaminations might happen during sampling and influence the results of gene mutation analysis. The relatively small size of the lesion might add to this problem. Besides, due to the rarity of pancreatic MiNEN, further molecular studies are required to explore the other subtypes of MiNEN and in morphologically “collision” neoplasms.

In conclusion, our case provides new insights into the morphological features and the genetic changes in a composite IPMN-NET. We also recommend a broader and clearer category of MiNEN in clinical work.

## Supplementary Information

**Additional file 1.**

## Data Availability

The original contributions presented in the study are included in the article/Supplementary Material. Further inquiries can be directed to the corresponding authors.

## Abbreviations

CgA: Chromogranin A
HE: Hematoxylin and eosin stain
IHC: Immunohistochemistry
INDEL: Insertion or deletion
IOPN: Intraductal oncocytic papillary neoplasm
IPMN: Intraductal papillary mucinous neoplasm
ITPN: Intraductal tubulopapillary neoplasm
LOH: Loss of heterozygosity
MANEC: Mixed adenoneuroendocrine carcinoma
MANET: Mixed adenoneuroendocrine tumor
Mb: Megabase
MCN: Mucinous cystic neoplasm
MiNEN: Mixed neuroendocrine-non-neuroendocrine neoplasm
*MLL3*: Mixed-lineage leukemia protein 3
NEN: Neuroendocrine neoplasm
NET: Neuroendocrine tumor
NGS: Next-generation sequencing
*PCK1*: Phosphoenolpyruvate carboxykinase 1
SNV: Single nucleotide variant
Syn: Synaptophysin
TMB: Tumor mutation burden
WHO: World Health Organization
